# Innovative Polyelectrolyte Treatment to Flame-Retard Wood

**DOI:** 10.3390/polym13172884

**Published:** 2021-08-27

**Authors:** Marie Soula, Fabienne Samyn, Sophie Duquesne, Véronic Landry

**Affiliations:** 1 Wood and Forest Sciences Department, Faculty of Forestry, Geography and Geomatics, Université Laval, 2405 Rue de la Terrasse, Quebec City, QC G1V 0A6, Canada; marie.soula.1@ulaval.ca; 2 NSERC Canlak Industrial Research Chair in Interior Wood-Product Finishes (CRIF), Université Laval, 2425 Rue de l’Université, Québec City, QC G1V 0A6, Canada; 3 CNRS, INRAE, Centrale Lille, UMR 8207-UMET-Unité Matériaux et Transformations, University Lille, F-59000 Lille, France; fabienne.samyn@univ-lille.fr (F.S.); sophie.duquesne@centralelille.fr (S.D.)

**Keywords:** fire-retardancy, polyelectrolyte complex, wood protection

## Abstract

Fire protection has been a major challenge in wood construction for many years, mainly due to the high flame spread risk associated with wood flooring. Wood fire-retardancy is framed by two main axes: coating and bulk impregnation. There is a growing need for economically and environmentally friendly alternatives. The study of polyelectrolyte complexes (PECs) for wood substrates is in its infancy, but PECs’ versatility and eco-friendly character are already recognized for fabric fire-retardancy fabrics. In this study, a new approach to PEC characterization is proposed. First, PECs, which consist of polyethyleneimine and sodium phytate, were chemically and thermally characterized to select the most promising systems. Then, yellow birch (*Betula alleghaniensis Britt.*) was surface-impregnated under reduced pressure with the two PECs identified as the best options. Overall, wood fire-retardancy was improved with a low weight gain of 2 wt.% without increasing water uptake.

## 1. Introduction

Biophilia is a concept that was introduced in the 1980s by Edward O. Wilson [1] and defines humans’ innate tendency to love nature. When applied to architecture, biophilia refers to a strategy that reconnects people with the natural environment by creating wide openings onto nature or by incorporating natural elements [2]. Studies have proven the positive impact of this strategy on health, with reduced stress and increased productivity [3]. In this scope, wood interior finishing is a major tool. Wood is a widely available, durable, and easy to process material. However, it is also highly combustible, leading to major fire safety concerns (notably in terms of flame spread) [4]. Regulations limit the use of wood interior finishing in critical areas such as emergency exits, corridors, and stairways in high-rise buildings [5]. Improving wood’s fire behaviour is therefore necessary to increase the presence of wood in buildings.

Much research has been carried out to understand and develop wood fire retardancy (FR). Four main strategies have been reported [6]. Firstly, condensed phase action relies on catalysing wood degradation to increase char formation and reduce the volatilization of flammable gases. Secondly, gas-phase action aims to disturb gas-phase reactions by diluting radicals and/or flammable gases with non-flammable volatile, as water vapor, ammonia or carbon dioxide. The third strategy is based on a “thermic effect” that promotes endothermic reactions at the surface of the wood. The last strategy is to use coating. In this latter case, the key point is to create a protective layer that limits both access to oxygen in the pyrolysis zone and the release of flammable gases to the flame [6]. Until now, this has been the most widely used technology to protect wood against fire [7]. Different types of thin and thick FR coatings have been described and are commercially available.

Among the different fire protective coatings available, intumescent coatings are the most effective when applied thick [8,9]. However, they have several drawbacks that limit their market acceptance. First, obtaining a transparent intumescent coating is challenging, as high concentrations of mineral additives [10] are necessary for the coatings to be effective. Further, surface damage as well as the aging of painted wood can strongly affect the coating’s effectiveness due to degradation of the main components [11] and a decrease of coating adhesion to the substrate [12].

Layer-by-layer (LbL) coatings represent another type of coating that has been studied for many years and developed to protect various substrates [13,14], including wood [15]. It consists of alternating positive and negative layers made of polyelectrolytes or nanoparticles, among others [16], to form a thin protective coating on the surface of the material. Barriers of this type improve fire retardancy but their processing time related to the application and drying of each layer is prohibitive for industrial applications. For example, an LbL coating made of sodium phytate and nano-TiO_2_-ZnO maximum effectiveness of 10 bilayers has been studied [17], but it requires 90 min of wood soaking and 1 h of oven drying for each layer. Different application approaches, such as spraying, have been developed to avoid this processing issue [18], but they are not industrially viable yet and improvements must be demonstrated. 

Promising one-pot alternatives to LbL coatings are currently in development, notably polyelectrolyte complexes (PECs) in water-based solutions [19]. PECs are versatile species consisting of a polyanion and a polycation [20]. Depending on the pH of the solution, they can be water-soluble, thereby enabling the deposition or can be in solid form [21]. Sodium phytate (SPA) is a high phosphorus compound derived from rice that has been widely used in nutrition [22], antimicrobial [23] and fire protection [24] research. Phosphorus compounds are known to catalyze cellulose dehydration, accelerating the formation of a protective char that inhibits thermal and gas exchange at the wood surface. SPA is highly water soluble, making it an interesting polyelectrolyte for fire retardancy. Its deposition by LbL application and sol-gel processes has been studied on various substrates [25,26,27,28,29,30,31], including wood [17,32]. Wood has also been impregnated with phytic acid to improve fire retardancy [33,34,35]. In 2019, a PEC of polyethyleneimine and sodium hexametaphosphate was used to treat softwood samples [19]. Samples were first soaked in a water-soluble PEC solution at a basic pH and then treated with an acid to make the PEC water-insoluble by protonation of polyethyleneimine’s (PEI’s) amine groups, leading to complexation between the two polyelectrolytes. A weight gain of only 6 wt.% was obtained after 70 min of treatment, leading to a 10% reduction in the peak heat release rate in cone calorimeter tests. This approach is very interesting, but again, the significant processing time is a limitation for process industrialization.

Regarding the processes used to impregnate wood, the vacuum-pressure process is one option to introduce FR solutions into wood lumens. With this technique, high weight gains of up to 100% can be obtained depending on the wood’s anatomy, the solution’s viscosity, and the molecular weight of the compounds in solution [36,37,38,39]. Impregnation using phosphorus-based compounds such as ammonium polyphosphate [40], guanyl urea phosphate [41] or organo-phosphorus compounds [42] provides an improvement of wood FR properties. However, it can be difficult and costly to impregnate timbers that have low permeability using this approach due to the need for non-continuous processes and drying time [43]. PEC deposition using a reduced pressure process, has already proven to be an interesting alternative to permit increased weight gain at shorter treatment times [44,45]. 

Building on the previously reported results, the flame retardancy provided by PECs formed from SPA and PEI is studied. The influence of the PEI:SPA ratio on the PECs’ thermal stability and hygroscopicity of the PEC is explored. Yellow birch (*Betula alleghaniensis Britt.*) samples were impregnated with promising PECs using a reduced-pressure surface impregnation process. Their performance on wood was compared, which confirmed that characterizing PECs independent of the wood is of interest. Treated wood’s properties have been studied by cone calorimetry, thermogravimetric analysis, and dynamic vapor sorption. 

## 2. Materials and Methods

**Figure 1:**
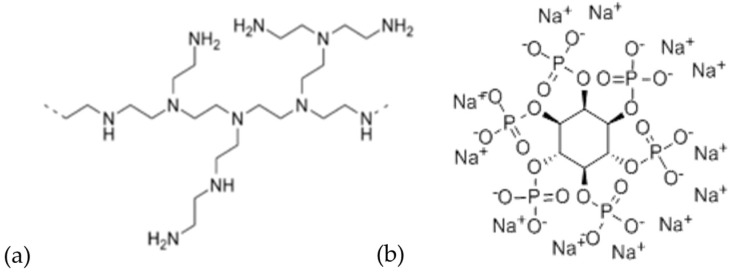
Branched polyethyleneimine (**a**) and sodium phytate (**b**).

Branched PEI (Mw ~ 25,000 g·mol^−1^) and SPA (from rice), both of which are displayed in Figure 1, hydrochloric acid (HCl, 37%), sodium hydroxide (NaOH, 97%) and citric acid monohydrate (CA, reagent grade, 98%) were purchased from Sigma-Aldrich (St. Louis, MO, USA) and used as received. Yellow birch (YB) boards measuring 60 × 10 × 0.4 cm^3^ were provided by Boa-Franc S.E.N.C. (Saint Georges, QC, Canada) and cut across the grain. All samples were free of defects and were sanded with a P-180 sandpaper grit and kept in a conditioning room (temperature = 20 °C, relative humidity (RH) = 40%) before use. For all tests, samples were selected from at least three different boards to consider wood variability.

### 2.1. Polyelectrolyte Solution Preparation

PEI and SPA solutions were prepared separately by dissolving PEI and SPA in deionized water. The pH of the PEI solutions was adjusted from 10.8 to 9 using 1 M HCl to keep the PEI in its unprotonated form (pKa = 8) [46]. The SPA solutions were kept at their natural pH of 3.5. Water-soluble PECs were prepared by mixing the PEI and SPA solutions at a 1:1 volume ratio. To form water-insoluble PECs, a 500 mM citric acid buffer was added. This buffer solution was prepared by dissolving citric acid in deionized water and adjusting the pH to 3.

### 2.2. Freeze Dried PEC Samples

To study the influence of the PEI:SPA ratio and acid treatment on the PECs’ formation and properties, different solutions were prepared with acidification (noted as “AC”) and without acidification. The details are summarized in Table 1. All solutions were immediately frozen for 24 h before being freeze-dried at 0.01 mbar with the collector temperature set at −80 °C using a Labconco FreeZone Plus 12 (Labconco, Kansas City, MO, USA) to analyze the PEC’s properties.

### 2.3. Surface Impregnation of Wood Samples

**Figure 2:**
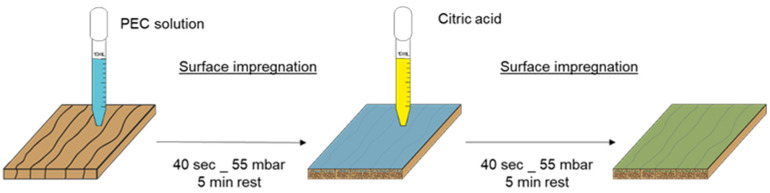
Schematic representation of PEC deposition method.

The selected PEC formulations were then used to treat wood samples. As presented in Figure 2, two impregnation steps were used to obtain the insoluble PEC treatments. First, the wood was impregnated with soluble PEC solutions. The solutions were applied to the wood surface so that they covered the entire surface (approx. 10 mL for 10 × 10 cm²: 2.5 mL for 5 × 5 cm^2^). Samples were placed in a reduced-pressure chamber, the pressure was reduced to 55 mbar for 40 s using a vacuum pump, and then the samples were left to absorb the solution for 5 min at atmospheric pressure. The excess solution was removed from the surface with a paper towel. The impregnation process (40 s reduced pressure + 5 min absorption time, removal of excess of solution) was then repeated with the citric acid solution (same volume as was used for the PEC). The treated samples were stored in a conditioning room for at least 10 days prior to any characterization to ensure the mass was stabilized.

### 2.4. Characterization

In this study, different characterizations were performed on the PEC alone and on the PEC-treated wood samples.

#### 2.4.1. PEC Characterization

Turbidity: The PECs’ stability in solution was evaluated using a Varian Cary 50 UV-Vis spectrophotometer (Agilent, Santa Clara, CA, USA). The wavelength selected for the analysis was 400 nm as neither PEI nor SPA absorb in solution at this wavelength [47,48]. Thus, any change in absorbance results only from the complexation of the polyelectrolyte. Solutions were prepared directly in the cell by first depositing 1 mL SPA solution and 1 mL PEI solution. Absorbance was first measured and noted as “_before”. The timer was started when adding 1 mL of the citric acid solution and absorbance was measured at 0 min, 5 min, 10 min, 30 min, and 60 min, which were noted as “_0”,”_5”,”_10”,”_30” and ”_60”, respectively.

Attenuated total reflectance Fourier transform infrared spectroscopy (ATR-FTIR): Fourier transform infrared spectroscopy (FTIR) was used to characterize the PECs before and after acid treatment. FTIR spectra were recorded on the freeze-dried PECs with an INVENIO R spectrometer (Bruker Optics Inc., Billerica, MA, USA) in the range of 4000–400 cm^−1^ in ATR mode with 64 scans and a resolution of 4 cm^−1^.

Thermogravimetric analysis (TGA): The thermal stability of the freeze-dried PEC samples was studied using a thermogravimetric analyzer from Mettler Toledo (model TGA 851e, Greifense, Switzerland). Samples weighing 5–10 mg were heated at 10 °C·min^−1^ from 25 °C to 800 °C under a nitrogen flow of 50 mL·s^−1^.

#### 2.4.2. Wood Sample Characterization

Weight gain (WG)**:** The weight gain of the treated wood was calculated according to Equation (1) with m_*i*_ as the mass before and m_*f*_ as the mass after impregnation.

(1) $$WG\;\left(wt.-\%\right)=100\times \frac{m_{f}-m_{i}}{m_{i}}$$

Densitometry: The density of reference and treated wood samples was measured using an X-ray densitometer from QMS, Model QDP-01X, (Quintex Measurement Systems Inc, Knoxville, TN, USA). Samples measuring 5 × 5 × 0.4 cm were used. The samples’ dimensions and weight were measured before scanning. Density was measured at an interval of 0.04 mm through the wood thickness.

Micro-X-ray fluorescence (micro-fluo-X): The PECs’ impregnation depth was evaluated by analyzing the phosphorus presence from top to bottom of the samples using an M4 TORNADO micro-fluorescence-X spectrometer (Bruker Nano GmbH, Berlin, Germany). The incident X-ray beam voltage was 15 kV. Mapping was built with a step of 20 µm and an exposure time of 10 ms. Five wood samples were prepared by microtome (Leica model HistoCore AUTOCUT, Buffalo Grove, IL, USA) cutting to ensure a smooth defect-free surface. Analysis was conducted on 0.4 × 0.4 × 0.4 cm samples retrieved from the centre of the densitometry samples.

Inductively coupled plasma mass spectrometry (ICP-MS): The remainder wood samples from the densitometry experiments were ground using a 200 ZM grinder with a 1 mm sieve (Retsch GmbH, Haan, Germany). ICP-MS was conducted by Bureau Veritas (Québec City, QC, Canada) using an argon plasma. The dry wood powder was digested by hot nitric acid.

Dynamic vapor sorption (DVS): Sorption data were collected on a DVS Adventure water vapour sorption analyzer (Surface Measurement Systems, Allentown, PA, USA) equipped with a microbalance of 0.1 μg resolution. A 4 × 4 × 4 mm sample retrieved from the centre of the densitometry sample was placed in a stainless-steel mesh basket suspended from the balance inside a separate chamber with controlled temperature and RH. The sample’s mass was continuously recorded. Samples were dried for at least 24 h at 103 °C before analysis. This first drying was followed by 2 h at 0% RH at 25 °C. Sample was then exposed to a series of RH steps from 0% to 90% RH, followed by the same series in reverse order. RH was changed when the sample’s mass variation was <0.002 wt.% for 5 min or after 6 h.

Cone calorimetry: The fire reaction of materials was characterized using a dual cone calorimeter (Fire Testing Technology, East Grinstead, West Sussex, England) following the ISO5660 standard [49]. The following characteristics parameters were obtained during testing: time to ignition (TTI), heat release rate (HRR), peak of heat release rate (pHRR), total heat release (THR), and mass loss rate (MLR) during the test. Each of the 10 × 10 × 0.4 cm horizontal wood samples previously stored in conditioning rooms (T = 20 °C and RH = 40%) were placed 2.5 cm below a conic heater and isolated at their back face by rock wool. The heating of the cone part was monitored to impose a specific irradiance of 50 kW m^−2^ at the top of the sample. Ignition was caused by an ignition source above the sample. The HRR was calculated from the quantity of oxygen released during the combustion and detected via a calibrated gas analyzer.

Optical microscopy: Cone calorimeter residues were observed using a VHX-7000 digital microscope (Keyence Co. Ltd., Osaka, Japan). Full ring lighting was used to enable dark field observation, which is preferred for heterogenous samples.

## 3. Results

### 3.1. PEC Characterization

#### 3.1.1. Fourier Transform Infrared Spectroscopy (FTIR)

**Figure 3:**
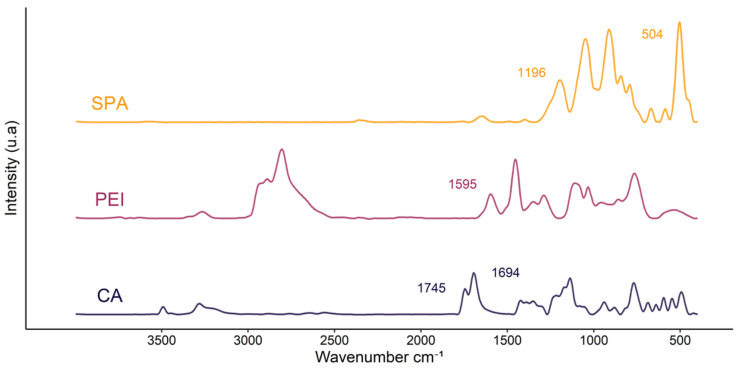
Spectra of sodium phytate (SPA), branched polyethyleneimine (PEI) andcitric acid (CA).

PEI, SPA and CA were characterized using FTIR and the spectra are displayed in Figure 3. For PEI, the band at 1595 cm^−1^ corresponds to the symmetric NH_2_ stretching vibration and peaks characteristic of CH_2_ vibration are also visible (at 2931, 2881, 2814, 1457 and 1346 cm^−1^) [50]. SPA is characterized by the two bands of PO_4_^3−^ at 1196 and 504 cm^−1^ and various C-O-P vibrations at 1048, 989, 911, 850 and 792 cm^−1^ [51]. Finally, CA’s major characteristic IR absorption bands are at 3492, 3448 and 3292 cm^−1^ for O-H stretching and 1745 and 1694 cm^−1^ for C=O stretching in the COOH groups [52].

**Figure 4:**
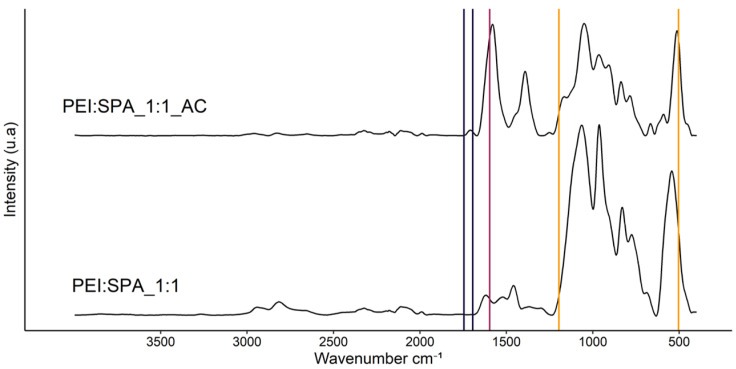
FTIR spectra of PEC_1:1 pre- and post-acidification.

Figure 4 compares the PEC made of PEI and SPA at a 1:1 ratio before and after acidification with CA. Before acidification, all characteristic peaks are shifted compared to the reference products. It is assumed that this shift resulted from the pH change from the original solution (PEI = 9, SPA = 3.4, PEI:SPA_1:1 = 9) and from the weak interaction between the two polyelectrolytes [53]. Acidification (PEI:SPA_1:1_AC = pH 5) led to further peak shifting and the appearance of new peaks around 1390 cm^−1^. Comparison of the pre- and post-acidification spectra confirms the change in interaction depending on the pH.

**Figure 5:**
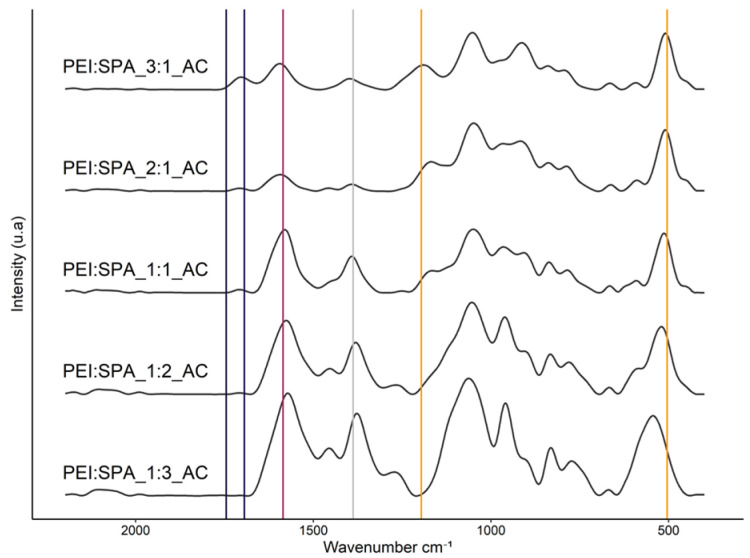
FTIR spectra of solutions of different PEI:SPA ratios after acidification.

The spectra of the acidified PECs of different ratios are compared in Figure 5. It is interesting to note that the peak shift around 1580 cm^−1^ is conditioned by the PEI:SPA ratio, as described in Table 2. The shift is greater when a higher proportion of SPA is used, which suggests that the polyelectrolytes’ interactions are stronger and the PECs are probably more likely to be stable in solution. In addition, a shift of the 504 cm^−1^ peak and the disappearance of the 1196 cm^−1^ one, corresponding to PO_4_^3−^, are also observed, which confirm greater interaction between PO_4_^3−^ and protonated NH_2_. Finally, as is described in the literature, a band appears around 1390 cm^−1^, resulting from the interaction between SPA and PEI [54].

#### 3.1.2. Turbidity

**Figure 6:**
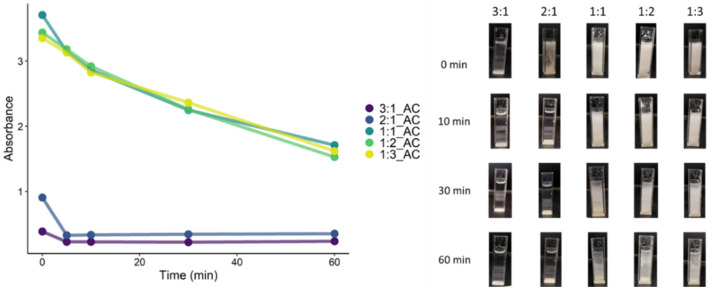
Absorbance over time for PECs after acid addition.

Complex formation as a function of pH as well as stability in water was studied by evaluating the solutions’ turbidity over time. Turbidity was evaluated by UV-visible absorbance, and the results are presented in Figure 6.

PECs can be either water-soluble or phase-separated (including turbid, coacervated or solid dispersed) depending on their pH, stoichiometry and ionic strength [55]. For PEI:SPA_2:1, PEI:SPA_1:1, PEI:SPA_1:2, and PEI:SPA_1:3, solid formation is observed immediately after adding citric acid (t = 0 min). This solid formation is associated with an absorbance increase (PEI:SPA_2:1 = 1, PEI:SPA_1:1 = 3, PEI:SPA_1:2 = 3, PEI:SPA_1:3 = 3). After 10 min, PEI:SPA_2:1 becomes soluble (PEI:SPA_2:1_10 = 0.2), indicating weak interactions between PEI and SPA [56]. For PEI:SPA_1:1, PEI:SPA_1:2, and PEI:SPA_1:3, absorbance also decreases over time (PEI:SPA_1:1_60, PEI:SPA_1:2, PEI:SPA_1:3 = 2). Their absorbance is still higher than the other ratios’ though indicating stronger interactions between polyelectrolytes. Also, pictures in Figure 6 indicate PEC coacervate formed, which can be described as a film more than individual particles [57].

#### 3.1.3. Thermal Stability

**Figure 7:**
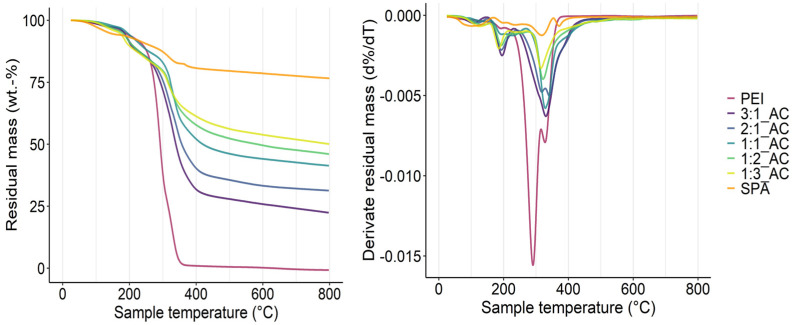
TG (**left**) and DTG (**right**) curves for PEI:SPA at different ratios under N_2_.

The thermal stability of PECs with different PEI:SPA ratios as well as PEI and SPA alone was investigated, and the results are presented in Figure 7, with the thermogravimetric analysis (TG) and the derivative thermogravimetric analysis (DTG), and Table 3.

PEI thermally decomposes in one step with a maximum decomposition rate at 290 °C and no residue at 800 °C. SPA is more thermally stable, with 76% residual mass at 800 °C. Five percent decomposition happens early (138 °C) for SPA, probably due its strong water absorption properties [58]. SPA decomposes in two steps around 260 °C and 320 °C. When studying PECs with different PEI:SPA ratios, it is worth noting that residual mass increases with the percentage of SPA in the complex. T_5%_ seems to have no clear tendency, and variation in the value may be due to residual water absorption. Early PEC decomposition might be advantageous for wood fire retardancy, as the catalysis of cellulose degradation is required to accelerate char formation. The half decomposition temperature increases with the percentage of SPA in the ratio. The remainder of the study focuses on PEI:SPA_1:2 and PEI:SPA_1:3, as they are more thermally stable and form water insoluble PECs.

### 3.2. Wood Samples Characterization

#### 3.2.1. Weight Gain and Depth of Impregnation

**Figure 8:**
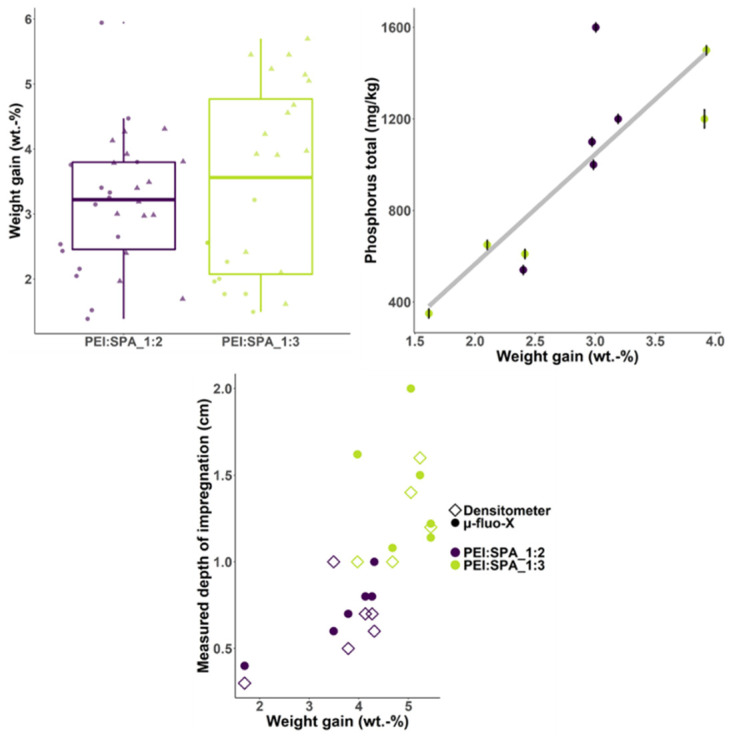
Summary of weight gain, phosphorus content, and depth of impregnation for treated samples.

Analyses were conducted to investigate the weight gain, depth of impregnation (densitometry and micro-fluo-X) and the final phosphorus content (ICP-MS) of treated wood samples. The results are summarized in Figure 8.

Weight gain is not significantly different for wood treated with PEI:SPA_1:2 versus PEI:SPA_1:3 and is around 3.5% for all samples (10 × 10 cm and 5 × 5 cm). Reduced-pressure surface impregnation occurs mostly by the small intervascular pits in the birch wood surface [59]. Weight gain is low (6% reported for whitewood stud) compared to that of similar treatment reported in the literature [19]. Weight gain variability in wood is strongly dependent on the wood species and is related to density variation, the proportion of sapwood/heartwood, and the presence of rings, among other factors [60,61,62,63]. Also, it must be noted that surface modification is strongly affected by surface quality and great care must be taken while sanding and storing samples to avoid surface oxidation [64,65]. This aspect was carefully considered in this study.

The ICP-MS experiments reveal that phosphorus content is proportional to weight gain, which was expected, and that the PEI:SPA ratio has no impact on phosphorus content.

The depth of impregnation was investigated using two techniques (densitometry and micro-fluo-X); they both have drawbacks that can alter results. Cupping is a known behaviour of surface densified wood that can cause a shift of the density gradient measured by densitometer [66], whereas micro-fluo-X provides a mapping of phosphorus in the wood depth. On the other hand, the quality of the microtomed surface is limiting in this latter measure as some contamination can occur. Despite this, these techniques provided consistent results, which provide a good approximation of impregnation depth. When plotting impregnation depth as a function of weight gain, it is clear that they are linked (the higher the weight gain, the higher the impregnation depth) and the PEI:SPA ratio has no influence on the impregnation depth.

#### 3.2.2. Cone Calorimetry

**Figure 9:**
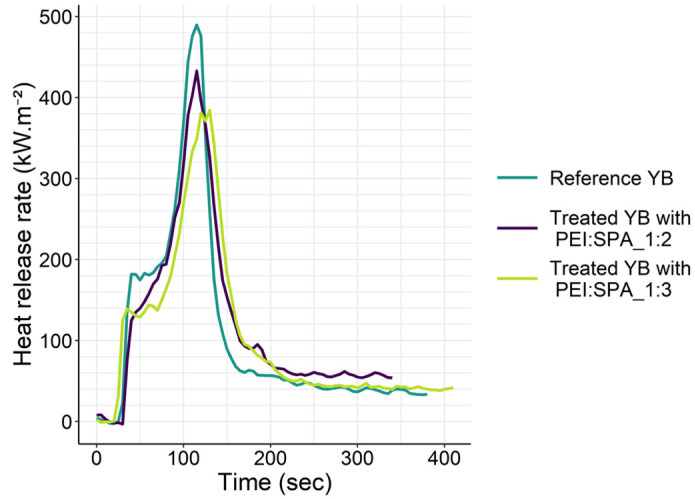
Heat release rate curves of yellow birch depending on the PEI:SPA ratio.

YB’s fire retardancy performance with different PEI:SPA ratios was evaluated by cone calorimeter and the results are presented in Figure 9 and Table 4. For untreated wood the first peak observed, just after ignition, characterizes primary pyrolysis that leads to the formation of a natural protective char. The pyrolysis process is thus slowed by the insulating char, leading to a plateau in the HRR curve. This protection ensures the temperature inside the wood is lower than at the surface of the char, which causes strong internal thermal stresses [67]. When these stresses are too strong, char cracking occurs and facilitates the release of combustible volatile species leading to a second peak of higher intensity. Treatment with either of the PECs changes the aspect of the curve. With PEI:SPA_1:2, the shoulder is considerably reduced, (Reference YB: 200 ± 10 kW·m^−2^, YB treated with PEI:SPA_1: 2: 130 ± 50 kW·m^−2^), which could be interpreted as more efficient char formation. The plateau is less defined than for the reference, however, which could signify the formation of a less thermally isolating char, corresponding to a thick intermediate non-charring material [68]. As for the YB treated with PEI:SPA_1:3, the shoulder is once again reduced. Contrary to with PEI:SPA_1:2, a plateau is visible and is clearly characteristic of a thermally isolating char inducing a reduction of the second HRR peak. Based on these results, a greater impregnation depth should help with the formation a thermally isolating char. PEI:SPA_1:3 has a lower TTI compared to the reference and the other treatment. Catalysis of the cellulose degradation by the phosphorus PEC is suspected. THR is not significantly different after treatment, whereas residue increases after treatment. When looking more in depth, it is interesting to note that YB treated with PEI:SPA_1:2 exhibits great p1HRR and p2HRR variability. It is supposed that HRR is linked to weight gain and great attention should be paid to sample preparation to control weight gain variation and ensure fire retardancy performance.

**Figure 10:**
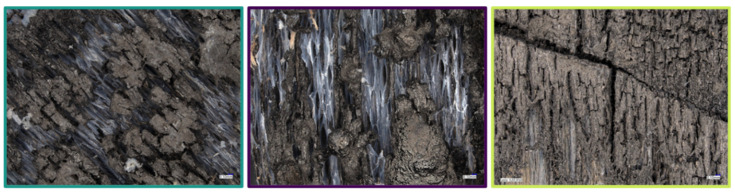
Observations of cone calorimeter residues: Reference (**left**), YB treated PEI: SPA 1:2 (**center**), and YB treated PEI: SPA 1:3 (**right**).

The cone calorimeter residues were observed using an optical microscope to better understand the charring phenomenon and images are displayed in Figure 10. The reference sample shows a carbonaceous char with the typical orthotropic pattern [67]. The fibrous structure of the wood is visible in the background, proving the natural protection the char provides. The treated samples display the same behaviour, as a thick char is visible above the degraded fibrous structure. However, in the YB treated with PEI:SPA_1:2 image, a glassy bubbly structure is clearly visible. The YB treated with PEI: SPA_1:3 also displays micro-bubbles, but the area observed also presents a similar pattern to the one observed in the reference sample. Similar phenomena were observed with phosphorus-based LbL systems used on cotton and have been attributed to micro-intumescence, a system acting in a condensed phase [69,70].

Classical approaches to wood FR have been widely studied, and pHRR reductions of more than 40% are now widespread. Wood has previously been vacuum pressure impregnated with clay [71], silica-based compounds [72], hydroxide-based compounds [73], and phosphorus-based compounds [74]. All of those treatments involve a reduced-pressure step to remove air from the wood and a high-pressure step where fire retardant substances are forced to impregnate the wood. It can also be followed by a curing step when fire retardant substances require a polymerization. These techniques result in weight gain up to 50 wt.% [75]. The reduced-pressure surface impregnation process described in this article does not permit such weight gain or, consequently, the same level of performance. However, this approach has to be considered as performance is satisfactory for material with only one side exposed to a fire and is more industrially viable. Comparison with LbL systems is difficult as, to the best of authors’ knowledge, no cone calorimeter measurements have been taken for them [17,32,76]. PEC deposition with PEI and sodium hexametaphosphate resulted in a 10% pHRR reduction following a one hour of treatment.

#### 3.2.3. Thermogravimetric Analysis

**Figure 11:**
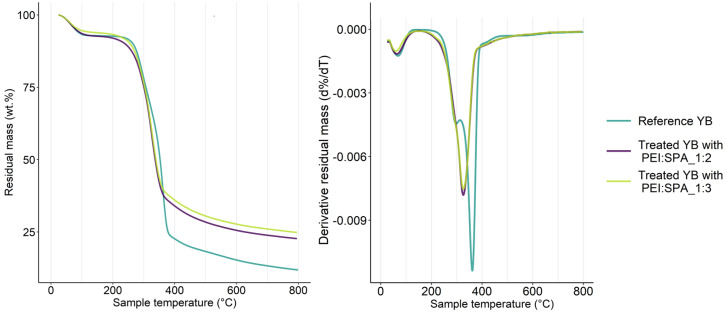
TG (**left**) and DTG (**right**) curves for reference YB and treated YB with different PEC.

A thermogravimetric analysis of the reference YB and YB treated with the two different PECs was conducted to understand the PECs’ contribution to wood fire-retardancy, and the results are presented in Figure 11 and Table 5.

Degradation of the reference YB is divided into three steps: drying, charring, and final decomposition [77]. First, weight loss occurs at around 100 °C and can be attributed to water removal. The charring stage, from 250 °C to 400 °C, involves decomposition of hemicellulose and cellulose into levoglucosan, the formation of char and the release of volatile species such as H_2_O, CO_2_, HCOOH and CH_3_COOH [78]. No volatile species are released anymore above 500 °C, and the residual mass corresponds to the thermally stable char [78]. Samples treated with PEI:SPA_1:2 and PEI:SPA_ 1:3 behave differently than the reference wood. According to DTG, the maximum degradation temperature is shifted down to a lower temperature with PEC treatment (Ref: 361 °C, PEI:SPA_1:2: 326 °C and PEI:SPA_1:3: 326 °C). Phosphorus compounds are widely known to catalyze cellulose degradation because of cellulose dehydration by phosphoric and polyphosphoric acids [79,80], which explains the shift mentioned. Treated YB with PEI:SPA_1:3 exhibits less desorption at 100 °C, which can be interpreted as a reduction in wood hygroscopicity after PEC treatment. These results show that PECs increase the wood’s thermal stability at high temperatures, as was previously assumed when discussing the cone calorimeter results.

#### 3.2.4. Dynamic Vapour Sorption

The hygroscopicity of flame retardant treated wood is a major concern, as it could induce severe dimensional stability and decay issues as well as important performance losses over a product’s service life [74].

**Figure 12:**
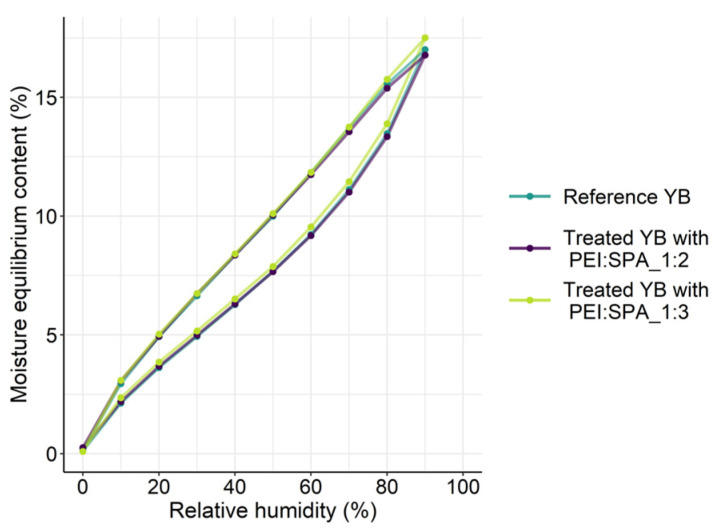
Isotherms for reference yellow birch and treated yellow birch with two different PEC ratios.

Isotherms for YB before and after treatment with the two PECs are presented in Figure 12. The reference YB presents the typical sigmoidal-shaped isotherm with hysteresis [81]. Water is first adsorbed as a monolayer in the internal surface of the wood’s cell walls. Then, from 15% RH, an adsorption polylayer forms in cell wall microcapillaries. Finally, above 70%, RH capillary condensation is predominant [61]. Both treated samples exhibit the same behaviour as the reference YB with a moisture equilibrium content of 17% at 90% RH. Phosphorus-based fire-retardant substances are usually known to increase wood hygroscopicity [82]. Treatments that result in low weight gain limit propensity of high hygroscopicity and ensure a longer service life for wood products.

## 4. Conclusions

Polyelectrolyte complexes consisting of polyethyleneimine and sodium phytate are investigated to develop fire-retardant wood. In the first part, analyses were carried out on different polyelectrolytes alone (not deposited on wood). Varying the ratio of the polyelectrolytes proved to have a strong impact on insoluble complex formation, thermal stability and hygroscopicity. The two most promising PECs (PEI:SPA_1:2 and PEI:SPA_1:3) were impregnated on the surface of yellow birch samples under reduced pressure. Weight gain and impregnation depth seem to be independent of the PEI:SPA ratio. Fire retardancy was evaluated using cone calorimetry, and PEI: SPA_1:3 displayed the best results with a reduction of the two pHRR with little weight gain. Residue observations suggested a micro-intumescence mechanism, which combines condensed and gaseous phase action. Finally, the treatments’ impact on hygroscopicity was studied, and surface impregnation with PEC seems to have no negative impact on the wood.

Overall, the preliminary study of PECs makes it possible to accelerate fire-retardant material development by limiting the amount of fire testing needed. The approach is particularly interesting for variable materials, which exhibit high intrinsic standard deviation. For instance, in wood, fire behaviour can be strongly impacted by the sample’s properties, such as cellulose content [77], crystallinity [83], density and moisture content [84]. Other important feature such as hygroscopicity were also pre-investigated, and the approach was as relevant for them as it is for fire-retardancy.

## Figures and Tables

**Table 1:** Summary of tested polyelectrolyte complex solutions.

Name	PEI (wt.%)	SPA (wt.%)
1:3 1:3_AC	7.5	22.5
1:2 1:2_AC	10	20
1:1 1:1_AC	15	15
2:1 2:1_AC	20	10
3:1 3:1_AC	22.5	7.5

**Table 2:** Peak shifting depending on the PEI:SPA ratio.

PEI:SPA Ratio	Peak Position ≈ 1580 cm^−1^	Peak Position ≈ 500 cm^−1^
3:1	1595 cm^−1^	509 cm^−1^
2:1	1593 cm^−1^	509 cm^−1^
1:1	1580 cm^−1^	513 cm^−1^
1:2	1576 cm^−1^	520 cm^−1^
1:3	1572 cm^−1^	544 cm^−1^

**Table 3:** TG and DTG curve results for different PEI:SPA ratios under N_2_.

	T_5%_ (°C)	T_50%_ (°C)	T_max.decomp_ (°C)	Residue at 800 °C (%)
PEI	187	292	290	0
3:1_AC	184	339	330	22.4
2:1_AC	183	357	341	31.3
1:1_AC	193	420	329	41.4
1:2_AC	178	584	321	46
1:3_AC	169	-	315	50.1
SPA	138	-	317	76.7

**Table 4:** Cone calorimeter results of yellow birch (virgin and treated with PEC at two ratio).

	Reference YB	YB Treated PEI:SPA _ 1:2	YB Treated PEI:SPA _ 1:3
Number of repetitions	12	12	7
WG (wt.%)		2.7 ± 0.9	2.0 ± 0.3
TTI (s)	31 ± 2	32 ± 6	27 ± 3
Shoulder HRR (kW·m^−2^)	200 ± 10	130 ± 50	140 ± 10
p2HRR (kW·m^−2^)	490 ± 30	440 ± 60	380 ± 20
THR (MJ·m^−2^)	44 ± 3	38 ± 4	41 ± 3
Residue (wt.%)	7 ± 2	12 ± 2	12 ± 4

**Table 5:** Results from TG and DTG curves for reference YB and YB treated with different PECs

	T_5%_ (°C)	T_50%_ (°C)	T_max.decomp_ (°C)	Residue at 800 °C (%)
Reference YB	79	353	361	11.9
YB treated PEI:SPA_1:2	80	337	326	22.7
YB treated PEI:SPA_1:3	91	339	326	24.8

## Data Availability

The data presented in this study are available on request from the corresponding author.
